# Upregulation of P2Y_2_R, Active uPA, and PAI-1 Are Essential Components of Hantavirus Cardiopulmonary Syndrome

**DOI:** 10.3389/fcimb.2018.00169

**Published:** 2018-05-23

**Authors:** Virginie Bondu, Casey Bitting, Valerie L. Poland, Joshua A. Hanson, Michelle S. Harkins, Sarah Lathrop, Kurt B. Nolte, Daniel A. Lawrence, Tione Buranda

**Affiliations:** ^1^Department of Pathology, University of New Mexico School of Medicine, Albuquerque, NM, United States; ^2^Office of the Medical Investigator, University of New Mexico School of Medicine, Albuquerque, NM, United States; ^3^Division of Infectious Disease, Pulmonary, Critical Care, and Sleep, Department of Internal Medicine, School of Medicine, University of New Mexico, Albuquerque, NM, United States; ^4^Division of Cardiovascular Medicine, Department of Internal Medicine, University of Michigan Medical School, Ann Arbor, MI, United States

**Keywords:** HCPS, PAI-1, uPA, P2Y_2_R, integrin activation, zymography, immunohistochemistry, Hantatvirus cardiopulmonary syndrome

## Abstract

Sin Nombre virus (SNV) causes hantavirus cardiopulmonary pulmonary syndrome (HCPS) with the loss of pulmonary vascular endothelial integrity, and pulmonary edema without causing cytopathic effects on the vascular endothelium. HCPS is associated primarily with a dysregulated immune response. We previously found occult signs of hemostatic imbalance in the form of a sharp >30–100 fold increase in the expression of plasminogen activator inhibitor type 1 (PAI-1), in serial blood plasma draws of terminal stage-patients. However, the mechanism of the increase in PAI-1 remains unclear. PAI-1 is a primary inhibitor of fibrinolysis caused by tissue plasminogen activator (tPA) and urokinase plasminogen activator plasma (uPA). Here, we investigate factors that contribute to PAI-1 upregulation during HCPS. Using zymography, we found evidence of PAI-1-refractory uPA activity and no tPA activity in plasma samples drawn from HCPS patients. The sole prevalence of uPA activity suggested that severe inflammation drove PAI-1 activity. We have recently reported that the P2Y_2_ receptor (P2Y_2_R) mediates SNV infectivity by interacting in *cis* with β_3_ integrins, which activates the latter during infection. P2Y_2_R is a known effector for several biological processes relevant to HCPS pathogenesis, such as upregulation of tissue factor (TF), a primary initiator of the coagulation cascade, stimulating vascular permeability and leukocyte homing to sites of infection. As P2Y_2_R is prone to upregulation under conditions of inflammation, we compared the expression level of P2Y_2_R in formalin fixed tissues of HCPS decedents using a TaqMan assay and immunohistochemistry. Our TaqMan results show that the expression of P2Y_2_R is upregulated significantly in HCPS cases compared to non- HCPS controls (*P* < 0.001). Immunohistochemistry showed that lung macrophages were the primary reservoir of high and coincident localization of P2Y_2_R, uPA, PAI-1, and TF antigens. We also observed increased staining for SNV antigens in the same tissue segments where P2Y_2_R expression was upregulated. Conversely, sections of low P2Y_2_R expression showed weak manifestations of macrophages, SNV, PAI-1, and TF. Coincident localization of P2Y_2_R and PAI-1 on macrophage deposits suggests an inflammation-dependent mechanism of increasing pro-coagulant activity in HCPS in the absence of tissue injury.

## Introduction

Sin Nombre virus (SNV) causes a severe form of hantavirus cardiopulmonary syndrome (HCPS), with case fatality rates of 30–40%. No curative therapy exists, and treatment of the disease is supportive. The most severe cases require extracorporeal membrane oxygenation (ECMO) treatment; the survivor and non-survivor cases present clinically similar syndromes (Khan et al., 1996; Wernly et al., 2011). Detection of cytokine-secreting cells of myeloid and lymphoid origin in autopsy tissue of HCPS cases (Mori et al., 1999), has helped to advance the idea of a “cytokine storm” model of HCPS pathogenesis (Jenison et al., 1995; Nolte et al., 1995; Zaki et al., 1995; Mori et al., 1999; Koster et al., 2001). The premise of an immune-mediated mechanism of hantaviral infections is based on the presence of elevated levels of proinflammatory mediators in the systemic circulation of patients with acute hantaviral illness (Zaki et al., 1995; Mori et al., 1999; Kilpatrick et al., 2004; Hjelle, 2014; Safronetz et al., 2014; Bondu et al., 2015; Morzunov et al., 2015; Khaiboullina et al., 2017). SNV has a particular tropism for vascular endothelial cells in humans, which the virus infects without causing any visible cytopathic effects, yet causes the loss of endothelial cell barrier function (Nolte et al., 1995; Hjelle and Torres-Perez, 2010; Vaheri et al., 2013; Hjelle, 2014; Safronetz et al., 2014). Also, laboratory findings have revealed the occurrence of disseminated intravascular coagulopathy (DIC) in HCPS patients in the absence of clinical signs of bleeding, intravascular thrombosis and apparent damage to the vascular endothelium (Nolte et al., 1995; Zaki et al., 1995). We have recently shown that terminal HCPS patients experience a remarkable rise, in plasminogen activator inhibitor type 1 (PAI-1), of up to 100-fold above normal levels, within 48 h of death (Bondu et al., 2015). However, the cause for PAI-1 upregulation in this setting is not yet understood.

PAI-1 inhibits plasmin generation, by preventing tissue plasminogen activator (tPA), or urokinase-type plasminogen activator (uPA) from processing plasminogen to plasmin (Dellas and Loskutoff, 2005). tPA is primarily involved in the dissolution of fibrin in the circulation. uPA is a critical component of the inflammatory response that regulates leukocyte extravasation to inflamed tissue. uPA is synthesized as a single chain zymogen known as pro-uPA(Pannell and Gurewich, 1987) or scuPA (Higazi et al., 1996). scuPA is activated when it binds urokinase-type plasminogen activator receptor, (uPAR). Activated uPA cleaves the zymogen plasminogen, to produce the protease plasmin, which in turn converts pro-UPA to its active dimeric form (Kasai et al., 1985) or tcuPA (Higazi et al., 1996). PAI-1 inhibits uPA activity in plasma and most tissues by binding to both free tcuPA and tcuPAR-bound uPA. However, PAI-1 interacts poorly with the scuPA-uPAR complex (Higazi et al., 1996). For clarity in this manuscript, we will use pro-uPA to define inactive uPA only and scuPA and tcuPA to identify the active monomeric and dimeric forms. Impaired fibrinolysis resulting from high plasma concentration of PAI-1(Dellas and Loskutoff, 2005; Smith and Marshall, 2010) can lead to excessive fibrin accumulation within vessels and the focal hyaline membranes associated with HCPS (Zaki et al., 1995). Poor outcomes in a variety of diseases related to ischemic cardiovascular events, fibrosis, and cancer are linked to high levels of PAI-1 expression (Bajou et al., 2004; Dellas and Loskutoff, 2005; Vaughan, 2005; Czekay et al., 2011; Knudsen et al., 2011; Osterholzer et al., 2012).

Pathogenic hantaviruses recognize the PSI domains of low-affinity state α_v_β_3_ and α_Ilb_β_3_ integrins for host cell entry that leads to productive infection (Raymond et al., 2005). One long-held view is that hantaviruses bind to inactive integrins and lock them in an inactive state (Mackow and Gavrilovskaya, 2009; Dalrymple and Mackow, 2014; Mackow et al., 2014). However, recent single-molecule atomic force microscopy experiments from our lab have shown that SNV engagement of the integrin PSI domain induces higher affinity for its cognate RGD ligands (Bondu et al., 2017). We further demonstrated that bent conformation low-affinity β_3_ integrins interact in *cis* with the G protein-coupled receptor P2Y_2_R via an RGD sequence in the first extracellular loop of P2Y_2_R (Bondu et al., 2017). As a result, hantavirus engagement of the integrin-P2Y_2_R complex stimulates force-dependent integrin *outside-in* signaling. Outside-in signaling requires a surface-anchored ligand, which can support traction force upon its exertion (Chen et al., 2017). In contrast, soluble ligands do not sustain force and therefore cannot support outside-in integrin signaling (Schürpf and Springer, 2011; Nordenfelt et al., 2016; Li and Springer, 2017). The *cis* binding to P2Y_2_R primarily provides the requisite scaffold for outside-in signaling (Bondu et al., 2017). The co-distribution of P2Y_2_Rs and β_3_ integrins, in host tissue, makes it likely that the cell biology associated with this interaction maybe be relevant to HCPS pathogenesis. The P2Y_2_R expression is upregulated under inflammatory conditions, which involve the release of IL-1β by macrophages (Hou et al., 2000; Tafani et al., 2011; Chen et al., 2013; Peterson et al., 2013; Englezou et al., 2015).

Our previous studies have shown significantly higher plasma concentrations of IL-1β in terminal HCPS cases compared to survivors (Bondu et al., 2015). One might expect the upregulation of P2Y_2_R expression to potentiate SNV infectivity due to its role as an effector of SNV induced integrin activation (Bondu et al., 2017). In this study, we tested the hypothesis that P2Y_2_R is significantly elevated in lung tissue of HCPS decedents compared to non-HCPS cases. The premise of this study is that inflammation during HCPS drives an upregulation of P2Y_2_R expression. P2Y_2_R potentiates expression of tissue factor, the primary initiator of the coagulation cascade (Teesalu et al., 2004). Thus, its upregulation might promote a procoagulant state during HCPS. Using formalin-fixed, paraffin embedded (FFPE) autopsy tissues from 22 HCPS decedents we obtained from the State of New Mexico Office of the Medical Investigator (OMI), we studied the expression of P2Y_2_R relative to non-HCPS control samples using TaqMan and immunohistochemistry.

## Materials and methods

### Patient samples

We analyzed for the expression of P2Y_2_R and proteases in tissues from 22 (11 female) HCPS subjects whose deaths were investigated by the State of New Mexico OMI between 1993 and 2014. The HCPS samples were age and sex- matched to control samples [17 self-inflicted gunshot wound (GSW)], and 15 pneumonia (lobar pneumonia, bronchopneumonia, and pneumococcal). It is important to note that a significant number of the pneumonia cases had various comorbidities such as alcoholism, neurological, cardiovascular disease, kidney, and trauma. Tissues were formalin-fixed, paraffin-embedded (FFPE).

The study population for plasma samples comprised of *de-identified* subjects previously admitted to University of New Mexico Hospital (UNMH) for HCPS and used with IRB approval: UNM IRB#15-166 and UNM IRB#16-084. The subjects were stratified according to severity scores: Class I [9 mild cases, 3 female, age 32.3 ± 11.3, body mass index (BMI) 25.6 ± 5.9], Class II (11 cases, required ECMO intubation and survived, 6 female, age 38.8 ± 22.8 BMI 27.6 ± 6.2), Class III (5 terminal cases, 3 female, age 42 ± 20.5, BMI 30.2 ± 8).

### Antibodies

PAI-1 was detected with the H135 antibody, (Sc-8979, Santa Cruz). Tissue factor (TF) was detected by a rabbit monoclonal antibody (EPR8986 ab151748, Abcam). P2Y_2_R was detected with an H70 antibody (SC-20124, Santa Cruz) and polymorphonuclear (PMN) leukocytes were detected with antibodies against neutrophil elastase (ab68672, Abcam). SNV antigens were recognized by the addition of hyperimmune rabbit anti-SNV N protein (1:5,000). R2 antibodies targeting uPA antigens were a generous gift from Dr. Lars Engelholm (Finsen Laboratory /BRIC Copenhagen Biocenter Copenhagen, Denmark).

### P2Y_2_R expression levels

In all cases (22 HCPS, 17 GSW, and 15 pneumonia) routine hematoxylin and eosin (H&E) sections and autopsy reports were reviewed to confirm the identity of tissue segments in each slice. Because each case was associated with more than one FFPE block, a total of 186 sections were sliced. Also, each slice contained distinct tissue segments of one or more organs (e.g., lung, brain, kidney), nearly 500 tissue segments were analyzed. To measure the P2Y_2_R expression levels in the tissue segments, RNA was extracted from 10 μm sections of the FFPE blocks using the Qiagen RNeasy FFPE kit (cat# 73504) and the instructions therein. The extracted RNA was quantified with a Thermo Fisher Scientific Nanodrop spectrophotometer. We next synthesized DNA from the sample RNA templates by RT-PCR using random hexamers (Invitrogen, P/N 100026484) and M-MLV reverse transcriptase (Invitrogen, ca# 28025-013). P2Y_2_R gene expression levels in the DNA samples were measured in triplicate, using the TaqMan gene expression assay comprising of a Fast Universal PCR Master Mix from Applied Biosystems (cat# 4352042) and a P2ry2 commercial primer probe from Applied Biosystems (Hs04176264_s1 P2ry2) following manufacturer instructions. We used a WT P2Y_2_R plasmid with known DNA concentration as a standard. We normalized the P2Y_2_R expression levels to the total RNA extracted from the embedded tissues.

### Immunohistochemistry

Immunohistochemistry analysis was performed at the UNM Department of Pathology, Human Tissue Repository, and Tissue Analysis Shared Resource (HTR-TASR) core facility.

Blocks of FFPE tissues were sectioned at 5 microns, and the parts mounted on charged (+) slides (Superfrost® Plus, StatLab Medical Products, Item #318). Slides were baked at 72°C for 60 min and then deparaffinized and rehydrated using reagents and following designated protocols for the automated Ventana Discovery immunohistochemistry slide stainer (Ventana Medical Systems, Tucson, AZ). The antibody was hand-applied and allowed to incubate for 60 minutes. Optimal dilutions of primary antibody and digestion conditions were determined by a series of preceding titration experiments using appropriate control tissues as needed (e.g., spleen for P2Y_2_R). Protein Atlas (https://www.proteinatlas.org/tissue) was used as a reference guide for antigen localization. The negative control was performed by omission of the primary antibody or tissue known for weak expression of antigen (e.g., non HCPS tissue was used to test specificity for SNV). Tissue sections were incubated with primary antibodies. Monoclonal antibodies against P2Y_2_R, SNV, PAI-1, TF and neutrophil elastase were used. The immobilized primary antibody was localized by subsequent application of species-specific secondary antibody, which was allowed to incubate for 16 min. Anti-HQ HRP (Ventana 760-4820) is applied and allowed to incubate for 16 min. Sections were then counterstained with DAB CM (Ventana 760-4304) in a working solution (H_2_O_2_ – four minutes, DAB CM – eight minutes, Copper – four minutes). For hematoxylin and eosin (H&E) staining, Hematoxylin (Ventana 760-2021) was applied and allowed to incubate for 4 min and then followed by Bluing solution (Ventana 760-2037) which was incubated for 4 min. Completed slides were removed from the Ventana Discovery, rinsed in a solution of Dawn dishwashing liquid and DI water (one drop of Dawn with 250 mL DI water). The slides were then cleared in 95% EtOH (10–20 dips), 100% EtOH (3 × 10–20 dips), and Xylene (3 × 10–20 dips). Slides were placed in cover-slips by removing one slide at a time from the Xylene, applying one drop of Permount® (Fisher Scientific, Item #SP15-500), and laying a glass coverslip over the tissue section.

### Measurements of total tPA, active tPA, and active uPA

Active tPA and uPA were measured by zymography as previously described (Teesalu et al., 2004). Human tPA total antigen assay ELISA kit (HPAITPAKT-COM) (Molecular Innovations, Novi, MI), was used to measure the expression of total tPA in HCPS samples, following a protocol supplied by the manufacturer (Molecular Innovations, Novi, MI, USA).

### Statistics

Data were transferred to an Excel spreadsheet and then analyzed using both SAS (Statistical Analysis Software) version 9.2 and GraphPad Prism. Spearman correlation coefficients were calculated for BMI and P2Y_2_R RNA expression. The average expression of P2Y_2_R in HCPS and controls in tissue samples and tPA in plasma samples were analyzed using two-way Analysis of Variance (ANOVA), with Dunnett's multiple comparison tests for pairwise comparisons. Linear regression models were used to assess variables associated with predicting average P2Y_2_R expression in the lung. *P* = 0.05 or less were considered statistically significant.

## Results

### Acute upregulation of PAI-1 is independent of tPA

**Figure 1:**
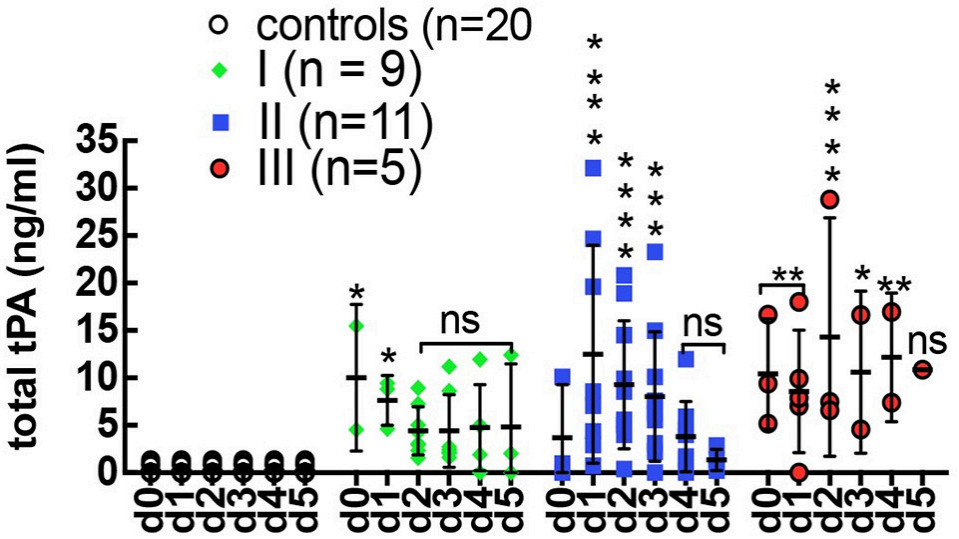
Expression total tPA in control (*n* = 30) healthy subjects and serial plasma samples from class I (*n* = 9), class II (*n* = 11), class III (*n* = 5) HCPS patients. Error bars represent ±SD of the mean among different cases on each day. Controls samples were measured on day 0 only and displayed on the graph for all days to allow for statistical analysis. Significant differences were found among the HCPS and controls early post admission but diminished with time Statistical significance was determined by two-way ANOVA Dunnett's multiple comparisons tests, ^*^P < 0.05, ^**^P < 0.01, ^***^P < 0.0001, and ^****^P < 0.0001.

In previous studies, we showed that PAI-1 increased dramatically at the onset of stage III HCPS by rising more than 100-fold above average levels compared to the mild class I (no change) and severe class II cases (5-fold increase, on average) (Bondu et al., 2015). We first determined whether the rise in PAI-1 was a response to increased tPA activity, by measuring the concentration of total tPA in longitudinal plasma samples (Figure 1). The data show the upregulation of tPA expression during HPCS relative to healthy controls. However, an increase in total tPA was not correlated with severity. In Table 1 we show tPA and PAI-1 expression data from the same samples in adjacent columns. We found no correlation between acute changes in PAI-1 and total tPA expression.

**Table 1:** Summary of available demographic and PAI-1 expression in class III patients.

**ID**	**Age/Sex**	**Draw date, Time**	**ECMO date, time**	**Death**	**WBC^1^**	**N#/L#/M# K/μl^2^**	**tPA ng/ml**	**PAI-1^3^ ng/ml**	**PAI-1 Δ-fold^4^**	**Active uPA^5^**
256	69/F	dy 1, 04:00	dy 1, 22:36	dy 4		Not available	7.0	1,229	61	Yes
		dy 2, 04:00			23.9	**13.1/6.7**/0.7	7.5	362	18	Yes
		dy 3, 04:01			16.4	**9.3/4.1/1.1**	4.6	220	11	Yes
260	63/F	dy 0, 23:31	n/a	dy 1	28.2	**23.0**/2.0/**1.7**	16.7	377.0	18	No
		dy 1, 4:22			34.7	Not available	0.0	1,169.5	58	No
280	30/M	dy 0,17:17	dy 1, 04:19	dy 3	7.4	5.4/0.5/0.4	9.4	62.0	3	Yes
		dy 1, 01:09			11.1	5.5/**4.1**(1.6)^a/^0.7	9.9	444.0	21	No
		dy 2, 04:24			18.9	**12.1/4.7**/0.2	6.5	2,769.5	132	Strong
281	37/M	dy 0,21:53	dy 1,15:32	dy 3	8.0	5.9/1.0/0.5	–	83.65	4	–
		dy 1,03:15			15.3	6.6**/6.7/**0.8	–	227	11	–
		dy 2,04:16			27.5	**9.1/12.7/1.7**	7.9	738.5	35	–
319	51/F	dy 0, 15:45	dy 1, 17:39	dy 21	18.1	**17.8**/0.9/**0.9**	5.2	187.5	9	–
		dy 1, 02:00			18.2	**15.7/**0.91/**1.28**	18.0	887.5	42	No
		dy 2, 03:00			19.6	**13.7**/2.4(1.4)^a/^**1.3**	28.8	77.50	4	Yes
		dy 3, 02:00			17.4	Not available	16.7	125.00	6	Yes
		dy 4, 04:00			17.5	**13.3**/1.8(1.4)^a^/0.0	17.0	95.5	5	Yes

1 *WBC reference range 4.0–11.0 × 10^3^/μl*.

2 *Absolute counts neutrophils (N#), lymphocytes (L#), and monocytes (M#). Bold numbers are out of normal range; N#: 1.8 −7.0 × 10^3^/μl, abs L#: 1.0–3.4 × 10^3^/μl, abs M#: 0.2–0.8 × 10^3^/μl. ^a^Values in parenthesis represent atypical lymphocytes (immunoblasts)*.

3 *PAI-1 expression in HCPS patients (256, 260, 280 and 281) was previously reported (Bondu et al., 2015), Case 319 data is was not previously reported*.

4 *Fold-increase, rounded to the nearest integer above the average expression of PAI-1 in humans (21 ng/ml)*.

**Figure 2:**
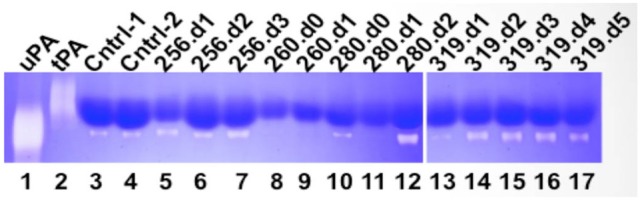
Qualitative zymography used to determine active tPA and uPA in plasma drawn from Class III HCPS cases (256, 260, 280, and 319). Coomassie blue stained zymogram reveals sites of proteolysis as white bands on a dark blue background. Data indicates active uPA in HCPS cases (note lanes 7, 12, 14, 15, 16, and 17). tPA activity was not detected. Although control samples indicate active uPA, the HCPS cases are notable, because of high levels of circulating PAI-1 in these subjects (Table 1).

5 *Refers to whether uPA activity is observed by zymography as shown in Figure 2*.

### uPA activity is atypically persistent under high PAI-1 expression in HCPS

We next examined the activity of tPA and uPA using a zymography assay (Figure 2). As expected there was no evidence of tPA activity in the zymograms. The zymogram of the control samples showed “housekeeping” protease activity (Cntrl −1, and Cntrl 2 in Figure 2). The plasma concentration of active PAI-1 in control samples is relatively low (21.2 ng/ml) (Bondu et al., 2015). Thus the intrinsic uPA activity in control samples was considered to be normal. The samples of each HCPS case indicated variable uPA activity. For context and clarity, it is useful to present the uPA results within the framework of changes in white blood cell (WBC) counts and active PAI-1 (Table 1).

256: On day 0, a previously healthy 69-year-old female was admitted to UNMH after developing acute nausea, 104°C fever, and vomiting after 3 days. A chest X-ray was consistent with pulmonary edema. She was placed on ECMO on day 1 (Table 1 for available blood draw times). The patient's condition required aggressive support including ventilator fraction of inspired oxygen (FiO_2_) of 100%. ECMO therapy was maximal, with two venous cannulas in the right internal jugular and right femoral vein with maximal extracorporeal membrane oxygenation flows between 4 and 4.5 liters per minute. Nevertheless, the patient presented signs of DIC and progressive multi-organ system failure and died on day 4. Her complete blood counts (CBC) with differential for day 2 and day 3 are shown in Table 1. The WBC count dropped from 23.9 K/μl to 16.4 K/μl respectively. Neutrophils and lymphocytes accounted for the decrease in WBC, but both remained above the normal range. Absolute monocyte counts increased, from 0.7 K/μl to 1.1 μl. The retrospective analysis of the patient's plasma found that PAI-1 was 61-fold above normal levels on day 1 sharply decreased to 18- and 11- fold above normal on the following 2 days. The decrease in PAI-1 was consistent with the fall in CBC counts. The zymogram shows that active uPA was present on each day ((256.d1-d3 in Figure 2).

260: On day 0 a 63-year-old female was transferred from a rural hospital in NM for suspected HCPS. According to the report, she originally presented to that hospital 5 days prior, for complaints of chest pain as well as fever and shortness of breath. She was diagnosed with a urinary tract infection while hospitalized. Two days later, a chest x-ray showed increasing pulmonary vascular congestion. Her WBC count increased from 4 to 12 K/μl, and she developed mild thrombocytopenia with initial CBC showing a platelet count of 156 K/μl. On admission (day 0) at UNMH, she was diagnosed with likely HPCS based on a 4/5 criteria for hantavirus on a preliminary evaluation of her peripheral blood smear, (Koster et al., 2001). However, the patient was not placed on ECMO. She remained unstable and continued to deteriorate despite maximal treatment with vasopressors and mechanical ventilation. She died the next morning (day 1 in Table 1). Her active PAI-1 was already elevated on day 0, (18 fold above average) and increased to 56-fold above average on day 1. Her WBC counts on day 0 were 28.2 K/μl and subsequently increased to 34.7 K/μl on day 1 (Table 1). Available CBC for day 0 indicated high neutrophilia, lymphocytes in the normal range and high monocytes. However, her uPA activity was below the detection limits of zymography on both days.

280: A 30-year-old male was transferred to UNMH with a history of acute weakness, dizziness, headache, myalgia, non-productive cough, nausea, vomiting, diarrhea, or abdominal pain (day 0). On day 1, he was placed on ECMO due to hypoxia. His ECMO course was complicated, requiring multiple vasopressors to maintain adequate cardiac output and ECMO flow. On day 2 he developed severe pulmonary edema. He developed multiple organ system failure including liver and renal failure. He died the morning of day 3. His WBC count increased from 7.4 to 18.1 K in 3 days. The CBC indicated that the increase in WBC on day 1 was due to an eight-fold increase in lymphocyte counts and near-doubling of monocytes compared to day 0. However, on day 2, the neutrophil counts doubled, and monocytes decreased by a factor of three from the previous day. Examination of plasma samples taken on day 0, 1, and 3 shows that PAI-1 increased sharply from 3-, 21-, and 132 - fold above average (Table 1). Attendant uPA activity was low and qualitatively similar to the intrinsic activity of control samples on day 0 (280.d0 in Figure 2). uPA activity was below detection limits on day 1 (280.d1 in Figure 2). However, on day 2 (280.d2 in Figure 2), the zymogram indicated very robust uPA activity, despite the highest peak levels of active PAI-1 yet measured in our cohort.

281: A 39-year-old male patient was transferred to UNMH for evaluation and treatment on day 0 at 21:53. During the previous 2 days he developed fever, nausea, vomiting, as well as diarrhea and poor appetite. The morning of day 0, he developed a new onset of shortness of breath. He had low platelets of 42, which were down to 18–26 on admission to UNMH. He was put on ECMO the next day (day 1), the patient died of massive hemorrhage and shock, on day 3 in the morning. The patient's WBC counts appeared in the normal range on day 0, but increased on day 1 due to acute lymphocytosis (Table 1). Subsequently his lymphocytosis worsened, in addition to an upregulation of neutrophils and monocytes. The analysis of PAI-1 expression indicated sharp increases of PAI-1 concentration in correspondence to the rise in WBC. We did not have sufficient samples to measure uPA. However it is worth noting that this case had very similar characteristics (demographic, history of illness, and differential immune cell response) to 280.

319: A 51-year-old woman felt ill with body aches 4 days prior to admission to UNMH. On day −3 she came to UNMH with her husband who had also become sick and was subsequently admitted for hantavirus infection (Class I). On day −2 she developed subjective fevers, prompting blood tests for hantavirus and peripheral smear, at which time she had 0/5 criteria (Koster et al., 2001) and had a negative hantavirus antibody test. She was admitted to UNMH on day 0. On day 1 she developed hypoxia and was intubated, sedated, and paralyzed for ECMO. She was successfully weaned from ECMO on day 4. On day 7 the patient developed neurological complications post ECMO. A head CT showed diffuse cerebral, midbrain, and cerebellar edema representative of global hypoxia. The patient later died on day 21. A review of the CBC from day 0 to day 4 indicates little day-to-day changes in WBC counts (Table 1). Interestingly, absolute neutrophil counts dropped by 4 K during the first 2 days. However, the decrease in neutrophils was offset by increases in monocytes, lymphocytes and atypical lymphocytes (immunoblasts). It is important to note that neutrophilia was predominant throughout this period. PAI-1 expression showed a 5-day trend of 9-, 42-, 4-, 6-, and 5-fold expression above average normal range (Table 1). uPA activity was analyzed for samples collected on days 1–5. The uPA activity on day 1 appeared to be qualitatively lower than the intrinsic proteolytic activity of the control samples (319.d1 in Figure 2). From day 2 onwards uPA activity appeared robust (319.d2-d5 in Figure 2). It is notable that the persistence of uPA activity in 319 did not elicit an antagonistic upregulation of PAI-1, such as occurred in the other cases with a shorter histories and courses of HCPS illness as presented above.

### P2Y_2_R is significantly upregulated in lung tissue segments of HCPS

FFPE -preserved tissue samples from 22 HCPS and case controls were tested for P2Y_2_R expression using a TaqMan RT-PCR assay. The case controls consisted of GSW, and 15 cases of pneumonia. Table 2 summarizes the demographic data of the HCPS and case-control population. Lung tissue blocks of different organ segments were available in sufficient quantities for HCPS and case controls to allow for robust statistical analysis. However, non-HCPS cases had limited availability of kidney, heart, and liver tissue segments. Thus, our study will focus on the analysis of lung tissue only. We analyzed 97 distinct segments from 20 HCPS cases as there was no available lung tissue from two cases. Thirty nine separate lung segments and 21 distinct segments were available from seven and nine pneumonia and GSW case respectively.

**Table 2:** Summary of P2Y_2_R study HCPS decedent population.

**Type of Death**	**Number**	**Female^a^**	**Mean age ±SD^b^**	**Mean BMI ±SD^c^**	**Mean BMI cases vs. control (*p*)**
Cases (HCPS)	22	12	37 ± 18.16	30.4 ± 6.48	
All Controls	34	15	40.9 ± 17.2	26.66 ± 7.1	0.04^*^
Pneumonia	15	7	42.7 ± 16.7	27.79 ± 7.7	0.3
Gunshot Wound	17	8	39.6 ± 18.5	24.30 ± 4.8	0.002^**^

a *No statistically significant difference in gender by cause of death (p = 0.52)*.

b *No statistically significant difference in mean age of cases and controls (p = 0.42)*.

c *No statistically significant difference in the presence of obesity in cases and controls (p = 0.26)*.

* P < 0.05;

** P < 0.01

**Figure 3:**
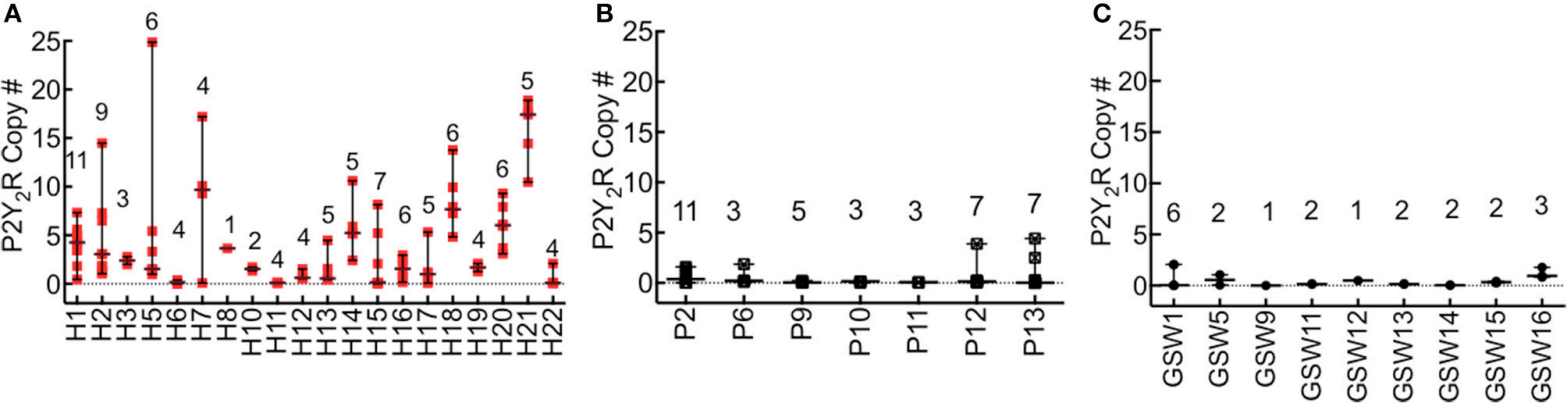
P2Y_2_R expression is significantly upregulated in HCPS compared to controls. RNA was extracted from lung tissues samples with the Qiagen RNeasy FFPE kit (cat# 73504) and quantified with a Thermo Fisher Scientific Nanodrop. P2Y_2_R expression levels were measured by the TaqMan assay using a WT P2Y_2_R plasmid as standard. P2Y_2_R copy #s were normalized to total RNA extracted from the embedded tissues. Each sample slice was measured in triplicate. **(A)** The plot of P2Y_2_R for each HCPS patient, shown on the x-axis as H1-H22. Each data point per patient represents a triplicate measurement of P2Y_2_R expression in a 10 μm slice cut from the FFPE blocks. The number of segments analyzed is shown for each case above the replicate data points (e.g., *n* = 11 for H1). Ninety-seven distinct segments from all cases were analyzed. Lung tissues from two decedents (H4, and H9) were not available. The error bars represent the median and range. For all HCPS samples, the minimum, median, maximum and mean values were 0.0055, 2.10, 24.88, and 4.1 ± 0.48 (SEM). **(B,C)** Plot of P2Y_2_R for pneumonia (P) and gunshot wound cases (GSW). The experimental conditions are similar to those described for HCPS. Cases for which no lung tissue samples were available were excluded from the graph. For all pneumonia tissue segment samples (*n* = 39), the minimum, median, maximum and mean values were 0.0005, 0.07, 4.4, 0.54 ± 0.16. For all GSW samples (*n* = 21), the minimum, median, maximum and mean values were 0.0052, 0.18, 2.4, and 0.46 ± 0.10 (SEM).

The difference in the expression of P2Y_2_R in FFPE lung tissue of HCPS and case controls was significant (all cases, *P* < 0.0001; GSW, 7-fold, *P* = 0.0001; pneumonia, 4 fold, *P* = 0.047). Figure 3 shows a summary plot of the P2Y_2_R expression in the available lung tissues of individual HCPS, pneumonia and GSW cases. Case-specific data points represent P2Y_2_R for distinct segments of lung tissue associated with each decedent. In HCPS samples, P2Y_2_R expression was variable among segments, (Figure 3A) whereas expression of P2Y_2_R in pneumonia (Figure 3B) and GSW (Figure 3C) cases tended to be homogeneous.

**Figure 4:**
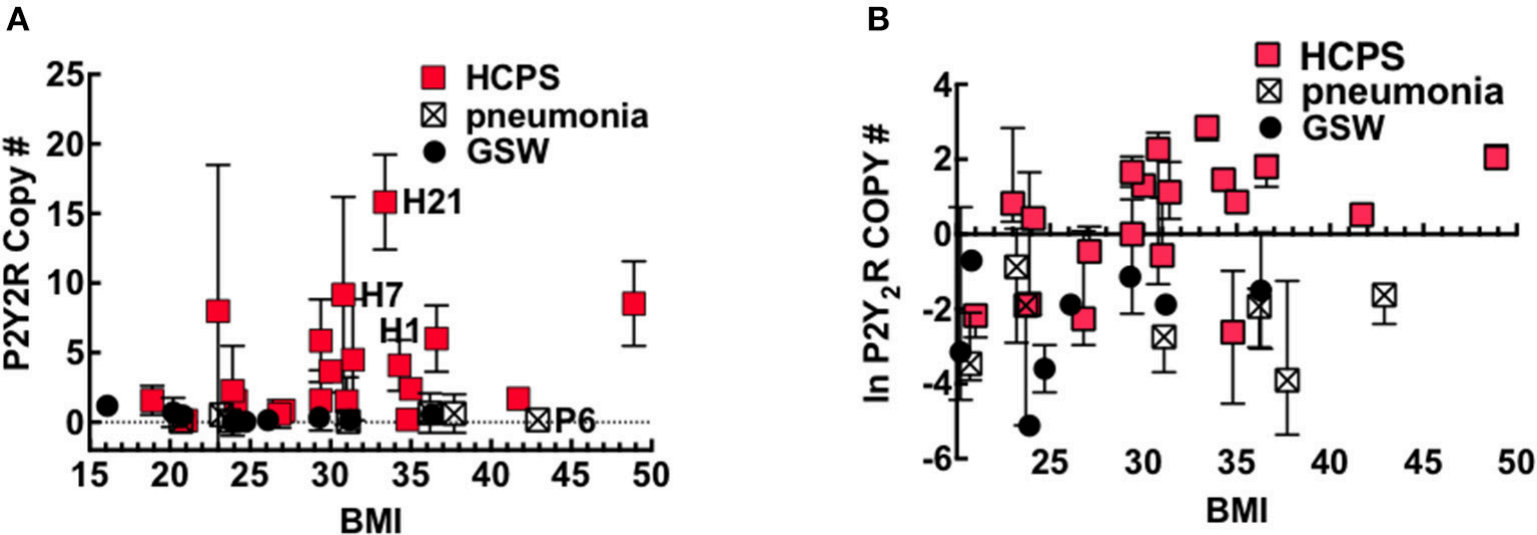
P2Y_2_R expression is significantly upregulated in HCPS compared to controls. Plots of **(A)** Mean and **(B)** Natural log-transformed values of P2Y_2_R copy numbers in 10 μm slices cut from the FFPE lung tissue blocks versus body mass index or BMI of Gunshot, pneumonia and HCPS cases. Error bars represent measurements of P2Y_2_R in slices from different segments of lung tissue from each case (indicated in Figure 3). Statistical analysis was performed on natural log-transformed data to approximate a normal distribution. See text for details.

Obesity is a factor in the pathogenesis of pulmonary diseases through mechanisms that involve adipose tissue that exert a low-level of systemic inflammation, also called meta-inflammation (Mancuso, 2010, 2013). BMI is a useful screening tool for the general health of an individual, with increasing BMI being associated with poor health. Because P2Y_2_R is upregulated under conditions of inflammation (Seye et al., 2002), we explored whether putative meta-inflammation in obesity predisposes people to increased P2Y_2_R expression. Figure 4A shows a plot of P2Y_2_R expressed in the lung tissue of HCPS, GSW and pneumonia cases vs. their BMI. The data were transformed to the natural log scale to approximate a normal distribution (Figure 4B).

The data were tested using Spearman correlation coefficients to determine whether BMI was a significant covariate for P2Y_2_R expression in HCPS. The results show a positive correlation between P2Y_2_R expression and increasing BMI; however, the correlation is not significant (Spearman coefficient *r* = 0.4, *P* = 0.08). In contrast the GSW (*r* = −0.35, *P* = 0.35) and pneumonia (*r* = 0.14, *P* = 0.76) that show no correlation. In linear regression modeling of average lung P2Y_2_R expression, the best predictor of average lung P2Y_2_R expression was type (case or control) (*p* = 0.002), followed by BMI (*p* = 0.16), when controlling for age and sex.

### High P2Y_2_R expression in lung sections is correlated to elevated SNV dissemination, macrophage deposits, uPA, TF, and PAI-1

**Figure 5:**
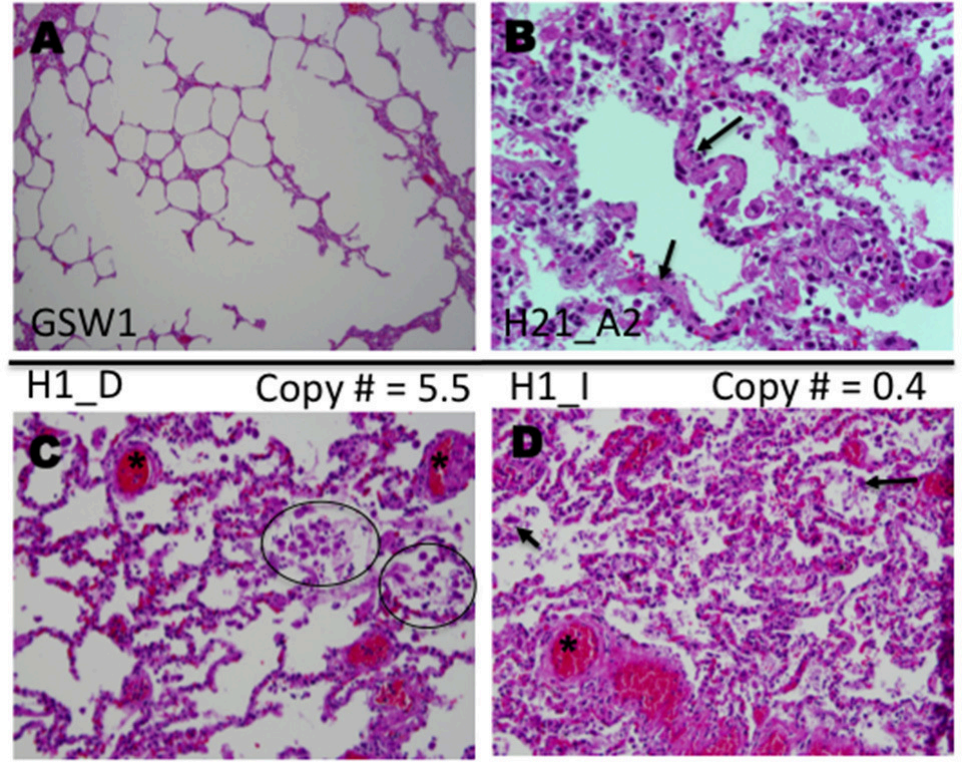
Histological analysis of lung samples of autopsied HPCS cases. Shown are representative sections of lungs stained with Hematoxylin and Eosin (H&E). **(A)** Sample of lung parenchyma of a gunshot wound decedent, showing open alveolar space (case GSW1). **(B)** Lung sample of an HCPS patient (case H21 Block A2) highlighting hyaline alveolar membranes (arrows), infiltrates of various immune cells immunoblasts, macrophages and neutrophils (few) (original magnification × 400). **(C)** Lung sample (case H1 Block D), highlighting focal deposits of macrophages (circles) and intraalveolar extravasated red blood cells (asterisk). **(D)** Lung sample (H1 case Block I) highlighting extensive congestion and intraalveolar edema with mild interstitial inflammation compared to (case H1 Block D) shown in **(C)**. The values of 5.5 and 0.4 in **(C**,**D)** respectively refer to P2Y_2_R RNA measured in the TaqMan assay (original magnifications for **C,D** × 200).

Our study benefits from the availability of detailed autopsy reports of the 22 HCPS decedents and associated publications (Nolte et al., 1995; Zaki et al., 1995; Mori et al., 1999). Histological examinations of the patient slides were consistent with previous findings (Nolte et al., 1995; Zaki et al., 1995; Mori et al., 1999). Healthy lung tissue is shown in Figure 5A. Characteristic histological features of HCPS include heavily congested lungs, interstitial inflammation composed of variable numbers of inflammatory cells (macrophages, immunoblasts, comparatively few neutrophils) in the alveoli or tissue, and hyaline membranes (Figures 5B–D) (Zaki et al., 1995; Mori et al., 1999). Examination of H&E slides of all subjects indicated a general trend where significant deposits of macrophages were correlated with relatively high expression of P2Y_2_R RNA. In a representative example herein, we examined the intraalveolar distribution of macrophages in different tissue segments of the same subject, with known high and low expression of P2Y_2_R (Figure 3, Case H1). Figure 5C shows prominent macrophage deposits in the tissue section sliced from a lung segment of Case H1 (FFPE block *D*) known to express elevated P2Y_2_R RNA (5.5 P2Y_2_R copy numbers). In contrast, a lung tissue segment expressing low levels of P2Y_2_R (block *I*, 0.4 P2Y_2_R copy numbers) shows scarce macrophage infiltration (Figure 5D).

**Figure 6:**
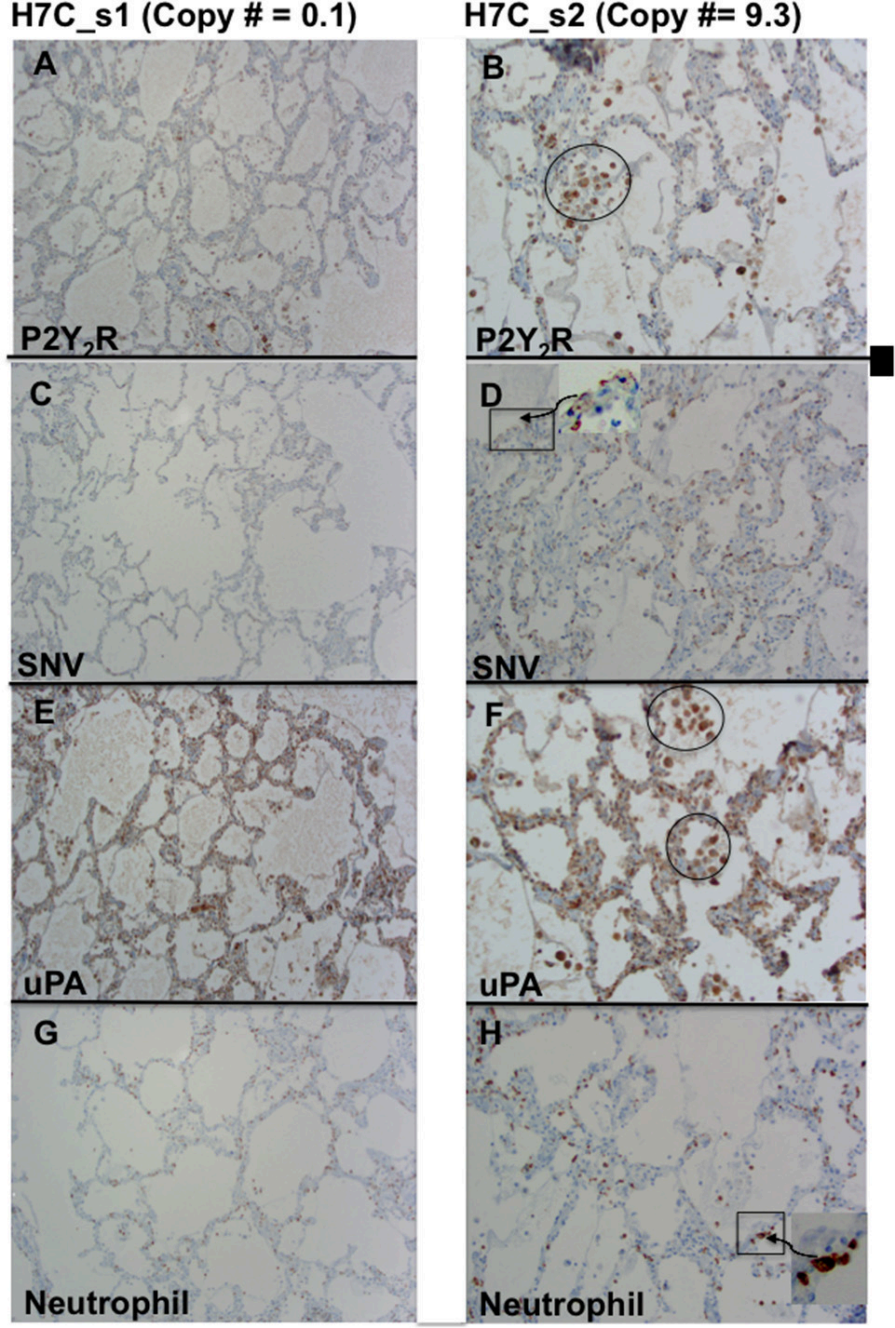
Comparison of serial sections from two different lung segments expressing low (s1) and high levels of P2Y_2_R (s2) from HCPS patient (case H7 Block C). **(A)** Weak staining for P2Y_2_R segment 1 corresponds to low P2Y_2_R copy numbers (0.1 TaqMan). **(B)** Relatively high P2Y_2_R copy numbers (9.3 TaqMan) is associated with a robust positive staining for P2Y_2_R in macrophages and endothelial cell-proximal type II pneumocytes. **(C)** Weak staining for SNV in segment 1. **(D)** Stronger positive staining for SNV in type II-pneumocytes and endothelial cells. **(E,F)** Comparative staining for uPA staining in *s1* and *s2* segments stronger staining in *s2* associated with higher macrophage deposits (circle). **(G,H)** Relative staining for neutrophil elastase in s1 and s2 indicates stronger staining for neutrophils related to higher P2Y_2_R (original magnification x200).

We next tested whether upregulation of P2Y_2_R RNA was correlated to increases in P2Y_2_R and SNV antigens, including the attendant immune response. We examined two lung segments, *s1* and *s2* (Figure 6), from the same FFPE tissue block C of decedent H7. As determined by the TaqMan assay, *s1* tissue sections were associated with very low, 0.1 P2Y_2_R, copy numbers, whereas *s2* segments displayed a > 90 fold higher level of P2Y_2_R expression. We thus carried out immunolocalization studies with antibodies reactive with P2Y_2_R, SNV, uPA, and neutrophil elastase in serial lung sections of the *s1* (0.1 P2Y_2_R copy numbers) and *s2* segments (9.3 P2Y_2_R copy numbers) (Figure 6). The conspicuous staining for P2Y_2_R antigens in *s2* sections was localized on noticeable macrophage deposits. In comparison, P2Y_2_R staining was low, and macrophage infiltration was rare in *s1* sections. Thus, the relative staining levels of P2Y_2_R antigens originating from *s2* and *s1* segments were in concordance with relative P2Y_2_R RNA expression (Figure 3). We found higher staining of SNV, uPA, and neutrophil elastase antigens in *s2* sections and conversely antigen staining for SNV and uPA and neutrophil elastase was universally weak in *s1* sections. SNV antigens were prominently localized on pneumocytes and endothelial cells (higher magnification images shown in Figure S1). uPA antigen staining was evident on macrophages and pneumocytes. Neutrophil elastase antigens were typically proximal to the alveolar walls.

**Figure 7:**
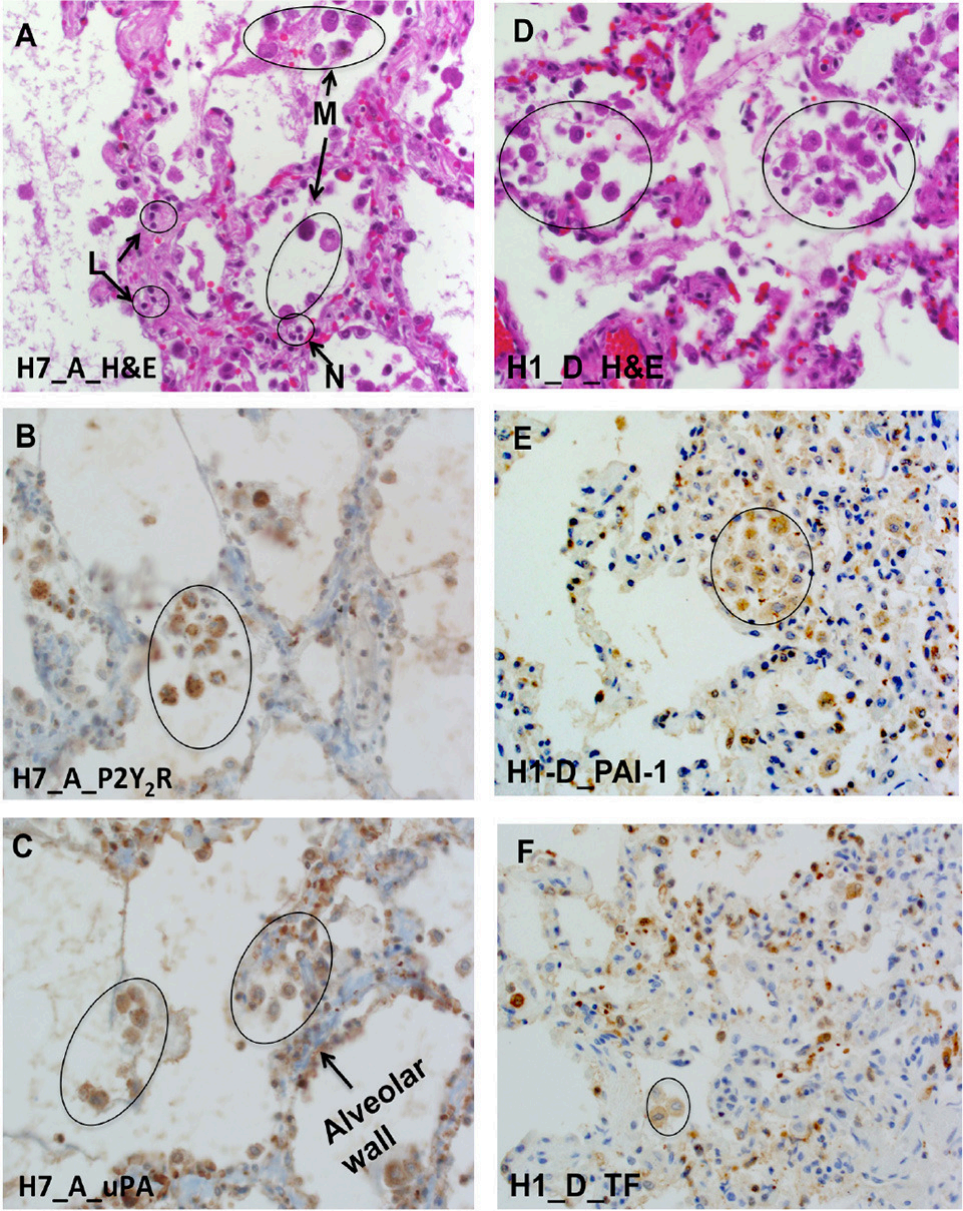
P2Y_2_R, uPA, PAI-1, and TF colocalize with macrophages in serial HCPS lung tissue sections. **(A)** Micrograph of lung tissue of HCPS patient (case H7 Block A) stained with hematoxylin and eosin (H&E) showing alveolar macrophages, neutrophils, and lymphocytes. **(B)** Immunohistochemical-detection of P2Y_2_R expression on macrophages and type 2 pneumocytes (H70 antibody: SC-20124). **(C)** Positive antigen staining for uPA in type II pneumocytes, hyaline membranes, alveolar macrophage (circles) (original magnification × 400). **(D)** H & E image of lung section of an HCPS patient (case H1 Block D) highlighting hyaline alveolar membranes, and macrophages in circles. **(E,F)** Serial Lung sections of case H1 Block D reveal cellular localization of PAI-1 and TF. PAI-1 mostly associated with macrophages and type II pneumocytes. PAI-1 was detected with the H135 antibody, Sc-8979 (Santa Cruz). TF staining was localized with alveolar macrophages or distributed in type II pneumocytes. Tissue factor (TF) was detected by a rabbit monoclonal antibody EPR8986 ab151748 (Abcam) (original magnification for all slides × 400).

Next, we performed immunolocalization studies with antibodies reactive with P2Y_2_R, uPA, tissue factor (TF), and PAI-1 antigens in sequential lung sections; Cases H7 and H1 are shown in Figure 7. As already indicated, macrophages were the source of the most intense colocalized antibody staining of P2Y_2_R, uPA, TF, and PAI-1 antigens. Weaker antibody staining was also present in pneumocytes and endothelial cells, albeit the weak signal could have been attenuated by the narrow width and surface area of the cells. Colocalization of PAI-1 and uPA on macrophages was notable. The results corroborated the zymography data, where uPA activity was shown to be refractory to elevated PAI-1 (cf. Figure 2).

**Figure 8:**
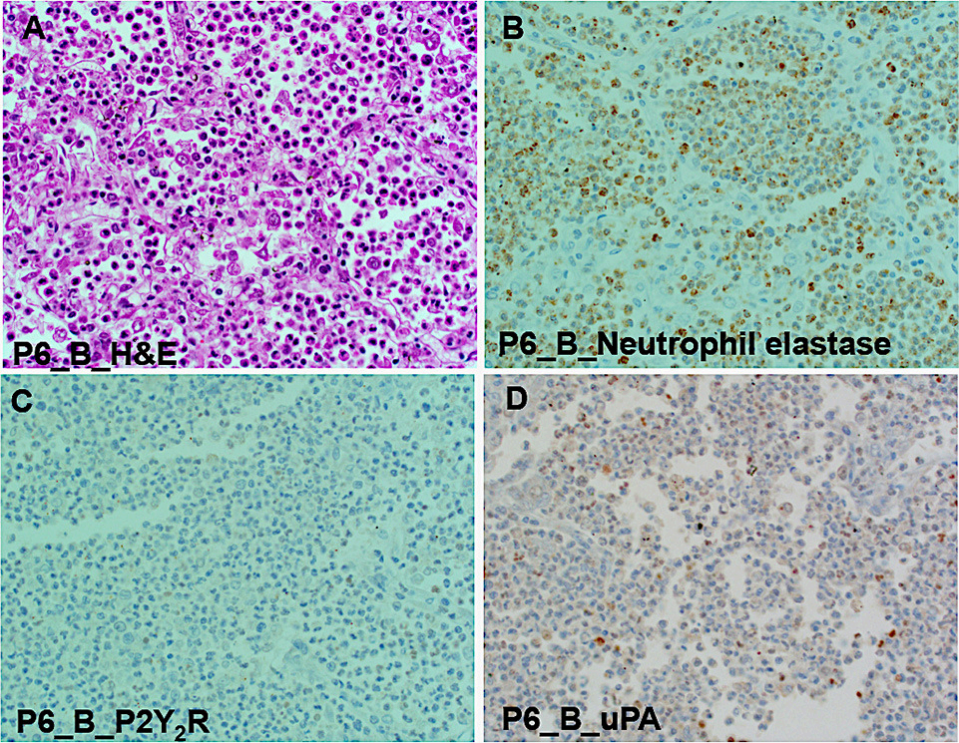
Immunohistologic examination of lobar pneumonia patient (case P6 Block B) shows strong staining for neutrophil elastase, but poor staining for P2Y_2_R and uPA. **(A)** Micrograph of lung tissue stained with hematoxylin and eosin (H&E) showing numerous neutrophils congest alveolar space and relatively few macrophages and lymphocytes. **(B)** Intense staining for polymorphonuclear (PMN) leukocytes with antibodies against neutrophil elastase (Abcam ab68672). **(C)** Weak staining for P2Y_2_R expression (H70 antibody: SC-20124), deficiency of P2Y_2_R is agreement with low P2Y_2_R (TaqMan measurement). **(D)** Weak staining for uPA is correlated to low macrophage expression.

We used immunohistochemistry to test for localization of P2Y_2_R, uPA, and neutrophils in tissue segments of the pneumonia patients. The H&E slide of subject P6 shows a prototypical lobar pneumonia case displaying many neutrophils and pockets of macrophage infiltrates (Figure 8A). Neutrophils elastase staining was robust (Figure 8B). The weak staining for P2Y_2_R (Figure 8C) is consistent with the TaqMan results on P2Y_2_R RNA expression. Also staining for uPA antigens in Figure 8D, was weak, though this finding could be related to the scarcity of macrophages. In sum, the immunohistochemical data were in agreement with TaqMan results.

## Discussion

*Cis*-complexes of β_3_ integrins and P2Y_2_R represent a novel pathway for rendering target cells permissive to hantavirus infection (Bondu et al., 2017). Our studies were designed to examine the possibility that HCPS induces the upregulation of P2Y_2_R expression. A related goal was to determine whether upregulation of P2Y_2_R in patient tissue potentiated SNV infectivity. According to the Protein Atlas (https://www.proteinatlas.org/tissue) P2Y_2_R expression is high in respiratory epithelial cells and low in pneumocytes and macrophages of the lungs of healthy subjects. Accordingly, we found weak expression levels of P2Y_2_R RNA and P2Y_2_R antigens in healthy lung tissue and pneumonia cases. However, in tissue from HCPS patients, P2Y_2_R RNA and P2Y_2_R antigens were significantly elevated in focal segments of various lung sections of HCPS cases.

**Figure 9:**
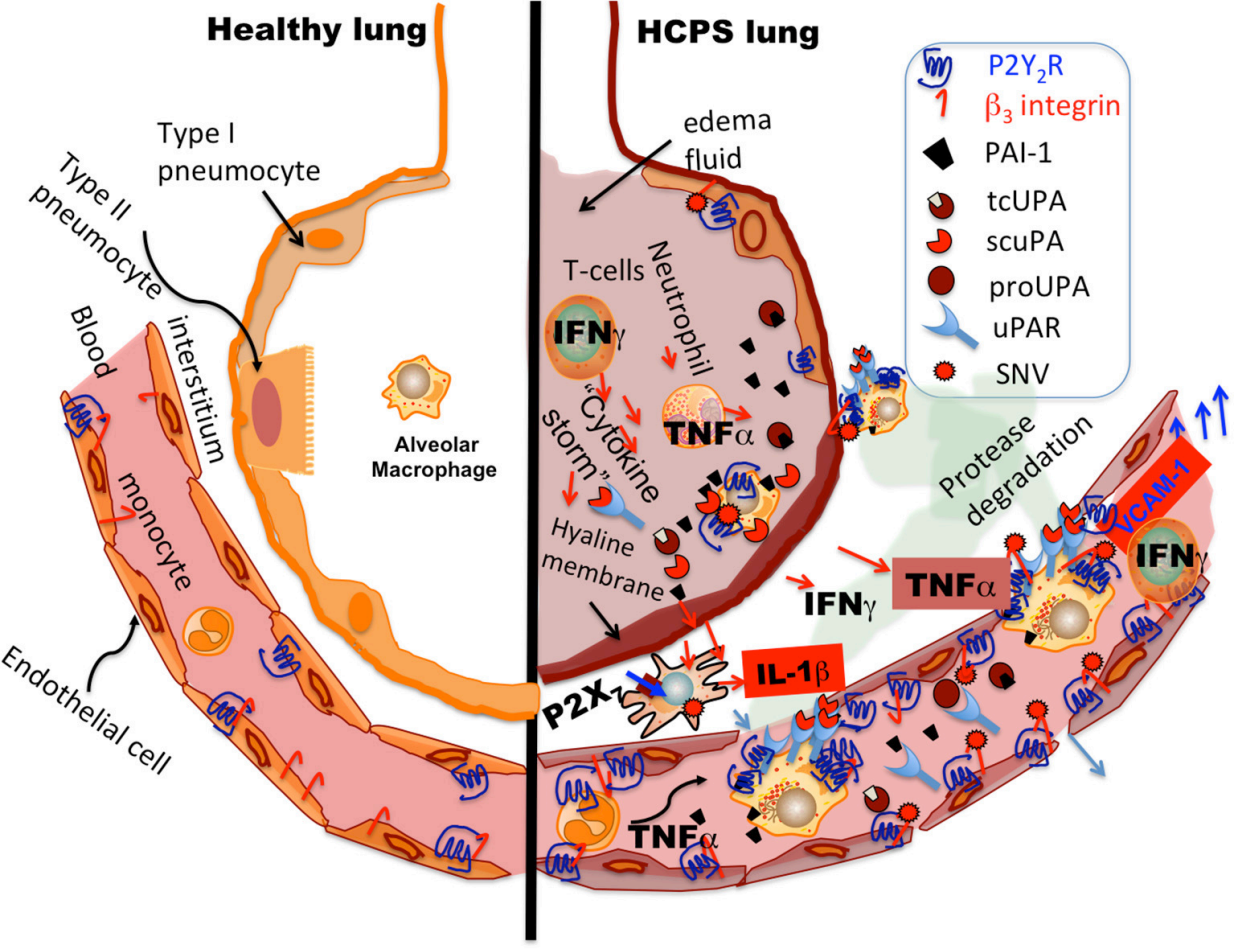
Model of the development of P2Y_2_R, scuPA, and PAI activity during HCPS (alveolus image was adapted from Kumar et al., 2015). Macrophages and dendritic cells recognize hantavirus after inhalation. Extracellular nucleotides increase under inflammatory conditions and activate P2 receptor family subtypes, including P2X_7_ and P2Y_2_R (Hechler and Gachet, 2015). Activation of P2X_7_ stimulates the release of IL-1β and upregulates P2Y_2_R in myeloid and endothelial cells. P2Y_2_R promotes, SNV infectivity, monocyte adherence to endothelial cells through VCAM-1 and contributes to chemotaxis. TNFα primarily by myeloid lineage cells and IFN-γ released by lymphocytes during HCPS (Mori et al., 1999; Safronetz et al., 2014) have been shown to upregulate soluble uPAR and expression of membrane-bound uPAR. Thus increased expression of uPAR and uPA is expected to enhance the proteolytic activity of chemotactic cells during HCPS. PAI-1 binds poorly to scuPA-uPAR complexes and might induce appositive feedback loop for increased protease activity.

Immunohistochemistry revealed simultaneous localization of P2Y_2_R, uPA, PAI-1, and TF antigens primarily on macrophages and pneumocytes in lung tissue. We observed intense focal staining of SNV antigens in the same tissue segments where strong P2Y_2_R antigen staining appeared. Conversely, macrophages, SNV, PAI-1, and TF were scarce in areas of low P2Y_2_R expression. The localized distribution of hantavirus antigens and “cytokine-storm” producing cells in the lungs of HPCS patients is consistent with earlier reports (Zaki et al., 1995; Mori et al., 1999) (Figure 9). Monocytes and macrophages are significant reservoirs of IL-1β production and secretion (Dinarello, 1996; Lopez-Castejon and Brough, 2011). IL-1β is known stimulate upregulation of P2Y_2_R expression (Seye et al., 2002). For this discussion, we have previously shown that IL-1β is significantly upregulated in terminal case HCPS patients compared to controls and survivors: (controls; 0.94 ± 0.76, Class; I 1.92 ± 0.63, Class II, 13.00 ± 2.03, Class III; 102.54 ± 32.80 pg/ml)(Bondu et al., 2015).

Macrophages and cells of monocytic lineage constitutively express both uPA and uPAR (Smith and Marshall, 2010). Exposure to inflammatory mediators enhances uPA and uPAR expression (Mondino and Blasi, 2004; Smith and Marshall, 2010). P2Y_2_R plays a significant role in monocyte (Santiago-Pérez et al., 2001; Seye et al., 2002; Liao et al., 2014) and neutrophil homing to injured or infected tissues (Chen et al., 2006). Also, itinerant immune cells (Kronlage et al., 2010; Desai and Leitinger, 2014; Idzko et al., 2014a,b) can release ATP from their leading edge to boost chemotactic signals and guide cell positioning by feedback signaling that involves P2Y_2_R (Kronlage et al., 2010). In response to a chemotactic gradient, monocytes/macrophages tightly cluster uPAR at the leading edge of migration, to focus uPA extracellular proteolytic activity during directed migration (Gyetko et al., 1994; Sitrin et al., 1994; Mondino and Blasi, 2004; Smith and Marshall, 2010) (Figure 9). Thus, it is noteworthy that we detected intense P2Y_2_R antigen staining on macrophage deposits in the lungs of HCPS patients.

### Multiple factors can lead to the upregulation of PAI-1 during HCPS

Endothelial cells regulate fibrinolysis, by secreting PAI-1 (Handt et al., 1996). Maladaptive responses to SNV infection foster a prothrombotic state, which is manifested by dysregulated increase of PAI-1(Bondu et al., 2015). PAI-1 inhibits uPA activity in plasma and most tissues by binding to the active two chain tcuPA and tcuPA-uPAR complexes but poorly interacting with the active single chain uPA (scuPA) (Higazi et al., 1996). Poor binding inhibition of PAI-1 to scuPA-uPAR complexes (Higazi et al., 1996) might contribute to the upregulation of the former, to favor increased equilibrium binding dynamics. Herein, our data suggest that PAI-1 is upregulated to counter refractory scuPA activity or exuberant increases in tcuPA depending on the case. This study is premised on the idea that severe inflammation causes dysregulated antagonism of uPA and PAI-1 in terminal HCPS. The expression of uPAR and uPAR-bound uPA is correlated with the invasive capacity of inflammatory cells. uPA bound to uPAR facilitates tissue invasion through the subendothelial extracellular matrix by catalyzing the conversion of membrane-bound plasminogen to plasmin (Smith and Marshall, 2010). Neutrophils comprise the most abundant of circulating white blood cells. Accordingly, neutrophils can outnumber lymphocytes, and monocytes by up to 2 orders of magnitude in reasonable health (Table 1). The relative populations of neutrophils, lymphocytes, and monocytes vary at different stages of infection. Neutrophils typically represent the first responders of the immune system, to cross the blood circulation vessel wall into tissue ahead of antigen-specific adaptive lymphoid cells (Mantovani et al., 2011; Kiermaier and Sixt, 2015; Lim et al., 2015). Migratory patterns and distribution properties of lymphocytes and monocytes are governed by the differential expression of inflammatory mediators (Rot and von Andrian, 2004). In this way, leukocytes sense and secrete mediators from similar cells or different cell types. Such a mechanism might allow different immune cell types to coordinate their serial emergence at a site of infection (Rot and von Andrian, 2004). A number of these inflammatory mediators are known to regulate uPA activity. For this discussion, the net uPA activity of mononuclear phagocytes is upregulated by monokines such as TNF-α and IL-1β (Kirchheimer and Remold, 1989). These inflammatory mediators are also chemoattractants of neutrophils, which in turn release the same inflammatory mediators. *In vitro* studies have shown that IFNγ released by lymphocytes upregulates the expression of uPAR, primarily in its membrane-associated form in monocytes, in contrast to TNF-α, which favors secretion of soluble uPAR (Gyetko et al., 1992; Sitrin et al., 1994). Along these lines, the net activity of uPA is modulated by the type and concentration of cytokines, released by different leukocytes. Stated differently, IFNγ primarily released by lymphocytes enables mononuclear phagocytes to focus uPA activity at the cell surface by upregulating the expression of membrane uPAR (Figure 9). Conversely, TNF-α enables the broader dispersion of proteolytic activity, by stimulating the secretion of soluble uPAR.

From our current limited dataset, it appears that the set point for dysregulated PAI-1 and uPA antagonism are established under variable immunological responses. Cases 280 and 281 present similar longitudinal distributions of neutrophils, lymphocytes, and monocytes, which are correlated with patterns of PAI-1 expression. On day 0, the circulating leukocyte subsets appeared to be in the normal range for 280–281. Interestingly, on day 2 both cases experienced lymphocytosis without any significant changes in neutrophil and monocyte populations. These relative changes in leukocyte populations induced substantial increases in active PAI-1. The rise in PAI-1 was sufficient to block uPA activity in available samples from case 280. Subsequent increases in neutrophil and lymphocytes on day 2 synergize to induce extremely high levels of PAI-1 expression. The combination of neutrophilia and lymphocytosis stimulated PAI-1-refractory uPA activity in 280 and 256. The leukocyte population of case 256 decreased longitudinally, however, the patient still presented with neutrophilia and lymphocytosis, which in turn stimulated active uPA despite high expression of active PAI-1. That lymphocytosis preceded neutrophilia as shown in case 280 and 281, is contrary to expectation (Rot and von Andrian, 2004). The cause for this observation remains to be determined. Conversely, uPA activity was inhibited in cases where 10-fold increases above normal in PAI-1, were solely associated with neutrophilia (case 260) or lymphocytosis (280, day 1) or monocytosis (319, day 1). Collectively these data suggest that variable distributions of leukocytes govern net uPA activity through coordinate regulation of secreted and receptor-bound uPA downstream leukocytes specific inflammatory mediators. This suggests that the expression of PAI-1 may be upregulated to counter uPA activity.

In our previous analysis of PAI-1 expression in pooled Class II samples, we found that an increase in PAI-1 of up to 10-fold above the normal range, on a given day was followed by a sharp and lasting drop in the expression of active PAI-1 in these patients (Bondu et al., 2015). Inflammation in Class II patients as measured by WBC tends to steadily decrease in subsequent days following hospital admission (unpublished data) and (Koster et al., 2001). Case 319's immune profile is more in concordance with Class II patients (Bondu et al., 2015). Unlike the cases who died from refractory HCPS during ECMO, Case 319 survived for more than 2 weeks after ECMO. After admission, 319's absolute neutrophil counts decreased after the acute upregulation of PAI-1, which coincided with monocytosis. Notably, the patient's lymphocytes did not exceed normal range, and thus PAI-1 derangement did not occur, even with persistent uPA activity, albeit to restrained PAI-1 activity. Collectively, the contrast in immunological response to HCPS of case 319 and the other 4 cases in the cohort, provide a potential turning point mechanism for mortality from refractory HCPS. Currently, an effort is underway to analyze cytokine, and protease activity in available individual case samples, and prospective samples, to determine critical immunological turning points that contribute to survival and mortality in HCPS.

The degree of vascular endothelial cell dysfunction induced by inflammation depends on the severity of endothelial cell activation (Pober and Sessa, 2007; Pober et al., 2009). HCPS patients who are not responsive to maximal medical treatment have predicted mortality of 100%. ECMO support reduces the mortality rate to 33% (Wernly et al., 2011). The criteria for refractive HCPS include a cardiac index of less than 2 L/min, refractory shock, and severe hypoxia as measured by the partial pressure of oxygen in the blood: fraction of inspired oxygen ratio (PaO_2_/FiO_2_) of < 60 (Wernly et al., 2011). Hypoxia, as occurs during refractory HCPS (Zaki et al., 1995; Bustamante et al., 1997; Wernly et al., 2011), is known to induce a prothrombotic phenotype (Pinsky, 1995; Pinsky et al., 1998; Liao et al., 2007). Our data show an acute increase in PAI-1 in blood sample draws taken before ECMO at which point the patients are known to experience severe hypoxia (Table 1). Oxygen deprivation drives vascular smooth muscle cells to express tissue factor, and mononuclear phagocytes to express tissue factor and PAI-1 (Hamer et al., 1981; Thake et al., 2004), which contemporaneously induces the formation of fibrin and protects it from fibrinolysis (Bertozzi et al., 1990; Olman et al., 1995; Pinsky et al., 1998). Impairment of fibrinolysis within lung tissues in favor of coagulation potentiates cytokines and chemokines involved in inflammation and chemotaxis (Ware et al., 2006).

## Summary

In summary, several factors contribute to the pathogenesis of refractory HCPS. Coordinated regulatory mechanisms of inflammatory mediators on protease activity might play a significant role in mortality. SNV infection appears to elicit a significantly more robust immune response from Class III cases than Class II, even though both classes experience refractory HCPS, with similar clinical syndromes (Wernly et al., 2011). HCPS severity and mortality are linked to relatively high levels of viremia (Terajima et al., 1999; Xiao et al., 2006; MacNeil et al., 2010; Yi et al., 2013). We propose the existence of an incidental interrelationship whereby positive feedback between a robust immunological response to SNV infection, upregulated P2Y_2_R expression and attendant uPA-orchestrated microphage mobilization to lung tissue, contribute to the dysregulated PAI-1 activity. As shown herein, increased expression of P2Y_2_R in lung tissue enhances SNV viral load. A potentiated immune response involving P2Y_2_R-directed mononuclear phagocytes that express tissue factor and PAI-1 establishes a positive feedback loop that increases opposing concentrations of active uPA and PAI-1. Limiting inflammation by inhibiting PAI-1 has been shown to be useful in cardiovascular diseases (Vaughan et al., 1997; Brown et al., 1999) and could be administered prophylactically to patients confirmed with HCPS. P2Y_2_R could also a be a potential target for discovery regarding the process by which it is upregulated during HCPS as well as the proclivity for promoting infectivity and a pro-coagulant state.

## Author contributions

VB and CB sample preparation, data collection, and analysis. VP identification of archived OMI tissue samples and cataloging and compilation of autopsy data. JH immunohistochemistry imaging and analysis. SL supervised VLP and performed statistical analysis on TaqMan data and manuscript editing. MH patient chart review, clinical insight, and summary of illness history. KN Chief Medical Officer, OMI, provided samples immunohistochemistry analysis, and manuscript editing. DL tPA and uPA analysis, manuscript editing. TB study coordinator, analyzed data and wrote the manuscript.

### Conflict of interest statement

The authors declare that the research was conducted in the absence of any commercial or financial relationships that could be construed as a potential conflict of interest.
