# Supplementary material for: Upregulation of P2Y2R, Active uPA, and PAI-1 Are Essential Components of Hantavirus Cardiopulmonary Syndrome
Source: Front Cell Infect Microbiol. 2018 May 23;8:169. doi: 10.3389/fcimb.2018.00169 (PMC6001748; doi:10.3389/fcimb.2018.00169)
Supplement: Supplementary file 1 [file Data_Sheet_1.docx]

Supplementary Material

**Upregulation of P2Y_2_R, active uPA and PAI-1 are essential components of hantavirus cardiopulmonary syndrome**

Virginie Bondu,^1^ Casey Bitting,^1^ Valerie Lynn Poland,^2^ Joshua A. Hanson,^1^ Michelle S. Harkins,^3^ Sarah Lathrop,^1,2^ Kurt B. Nolte,^1,2^ Daniel A. Lawrence^4^ and Tione Buranda^1*^

^1^Department of Pathology, University of New Mexico School of Medicine, Albuquerque, NM 87131, USA ^2^Office of the Medical Investigator, University of New Mexico School of Medicine, Albuquerque, NM 87131, USA ^3^Division of Infectious Disease, Pulmonary, Critical Care, and Sleep, Department of Internal Medicine, School of Medicine, University of New Mexico, Albuquerque, NM, USA.^4^Department of Internal Medicine, Division of Cardiovascular Medicine, University of Michigan Medical School, Ann Arbor, MI 48109, USA

*** Correspondence:** Tione Buranda, 337C BRF, Dept of Pathology, University of New Mexico School of Medicine, 915 Camino de Salud, MSC 084630, Albuquerque NM 87131

[tburanda@salud.unm.edu](mailto:tburanda@salud.unm.edu)

**SUPPLEMENTARY FIGURE**


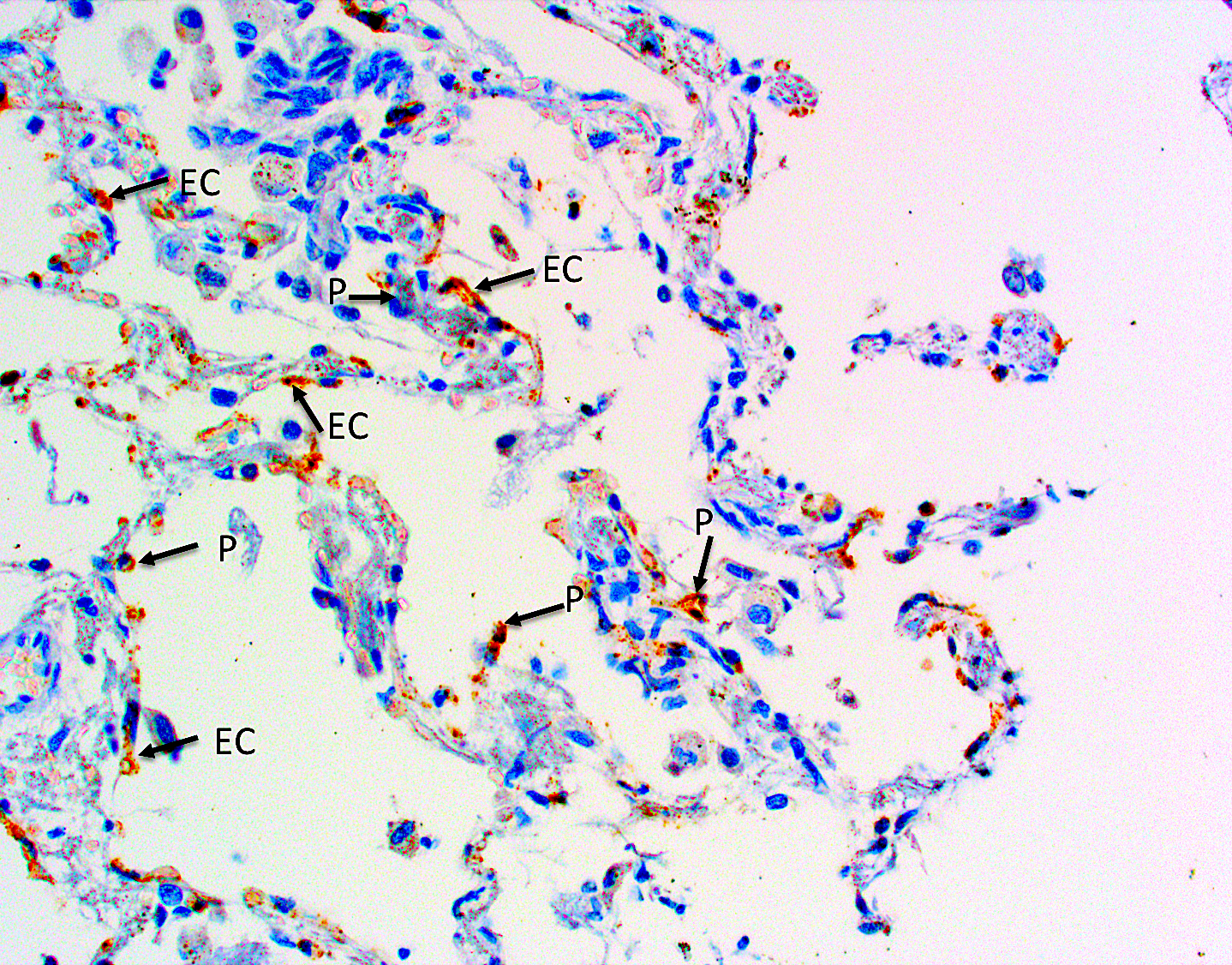


**Figure S1**. Immunohistologic examination of HCPS subject (case H7 Block A) shows staining for SNV in type II- pneumocytes (P) and endothelial cells (EC).
